# Developing Synthetic Parameters Using Frequency Band Ratios for Muscle Fatigue Analysis During Isometric Contractions by Using Shoulder Muscles

**DOI:** 10.3390/s25072191

**Published:** 2025-03-30

**Authors:** Ji Soo Park, Myung-Chul Jung, Jung Yong Kim, Seung-Min Mo

**Affiliations:** 1 Dasan University College, Ajou University, 206, World cup-ro, Yeongtong-gu, Suwon-si 16499, Gyeonggi-do, Republic of Korea; jspark21@ajou.ac.kr; 2 Department of Industrial Engineering, Ajou University, 206, World cup-ro, Yeongtong-gu, Suwon-si 16499, Gyeonggi-do, Republic of Korea; mcjung@ajou.ac.kr; 3 Department of Human Computer Interaction, Hanyang University, 55, Hanyangdeahak-ro, Sangnok-gu, Ansan-si 15588, Gyeonggi-do, Republic of Korea; jungkim@hanyang.ac.kr; 4 Department of Occupational Safety and Health Management, Osan University, 45 Cheonghak-ro, Osan-si 18119, Gyeonggi-do, Republic of Korea

**Keywords:** muscle fatigue, fatigue parameter, %MVC, frequency band ratio, electromyography

## Abstract

This study aimed to develop new parameters for electromyography (EMG)-based muscle fatigue assessments. First, various combinations of frequency band parameters, including the high-frequency band (HFB: >95 Hz), medium-frequency band (MFB: 46–95 Hz), and low-frequency band (LFB: 15–45 Hz), were assessed to evaluate the fatigue detection performance of individual parameters during isometric muscle contractions. The experimental design involved applying three force levels (30%, 40%, and 50% of the maximum voluntary contraction) and targeting three muscles (upper trapezius, mid-deltoid, and pectoralis major) due to their relevance in shoulder load postures associated with musculoskeletal disorders. A total of 15 participants were involved in this study. The effectiveness of each parameter was assessed through response sensitivity evaluations. Through these evaluations, we confirmed that the previously mentioned individual frequency bands, along with the proposed H/(M + L) frequency band, exhibited high statistical significance and sensitivity under various experimental conditions. Specifically, our findings demonstrated that the H/(M + L) frequency band effectively assessed fatigue levels with high sensitivity and accuracy at low force levels during static isometric contractions. Overall, these results are expected to improve the accuracy of evaluations of individual shoulder muscle fatigue, thereby reducing the risk of shoulder injuries.

## 1. Introduction

Electromyography (EMG) is primarily used to quantitatively assess muscle fatigue resulting from physical activities. EMG-based fatigue assessments generally involve two physiological aspects: First, a reduction in the conduction velocity of motor unit action potentials (MUAP), and second, an increase in motor unit (MU) synchronization by the central nervous system. In the study by Mesin et al. [1], the estimation of muscle fiber conduction velocity (ECV) was identified as a useful indicator for assessing peripheral fatigue, while fractal dimension (FD) was confirmed as a useful indicator for evaluating central fatigue. Another evaluation method involves analyzing time-series signals, such as assessing the zero-crossing rate (ZCR), root mean square (RMS), and the amplitude of muscle tension (AMT). It was concluded that ZCR and AMT are complementary indicators of muscle fatigue. Over the past few decades, researchers have extensively investigated the changes in various EMG parameters associated with fatigue during isometric muscle contractions, including conduction velocity, mean spectral frequency, median spectral frequency, RMS value, ZCR, AMT, and average rectified value.

However, RMS, which uses the amplitude of the EMG signal, has the drawback of being interpreted differently depending on the force level, as it evaluates fatigue relative to the magnitude of force [2]. ZCR has the disadvantage of being indistinguishable from noise during low-force muscle contractions [3]. To address these limitations, central frequency (MF) and mean power frequency (MPF), which use frequency analysis, have been employed. MF is sensitive to changes in muscle temperature, requiring caution, but it exhibits less variability compared to MPF [4]. MPF reflects physiological responses related to muscle contraction, making it useful for fatigue measurement, but it shows greater variability in results compared to MF. Additionally, while both MPF and MF represent information related to force, research has shown that MPF values become independent of force output when force levels exceed 25% of maximum voluntary contractions (MVCs) [5]. Allison and Fujiwara [6] noted that MPF, which uses frequency analysis methods, requires high sampling rates because signal non-stationarity and resolution are related to the sampling rate. To address the issue of signal non-stationarity, researchers have conducted studies to evaluate the stability of muscle signals and have suggested that maintaining a sampling rate of over 500 Hz is necessary for signal stability [7].

Recent studies have begun to use ratio parameters to address the issues associated with measurement methods that rely on EMG signal amplitude or frequency analysis [8]. Ratio parameters have been found to be useful for fatigue measurement in various studies [9,10,11,12]. In particular, the H/LFB (high/low frequency band) ratio is also effective in predicting muscle contraction duration and fatigue onset time [13]. Additionally, several innovative methods for electromyography (EMG)-based fatigue estimation have been introduced, including the ratio between spectral moments, continuous wavelet transform, time–frequency representations, autoregressive analysis, and signal entropy analysis [14,15]. In this context, measurements of frequency band ratios are advantageous as they do not require high sampling rates and are less affected by signal instabilities [12]. Specifically, the H/LFB ratio can predict muscle contraction duration and the time of fatigue onset [13]. Similarly, the L/H (low/high) frequency band ratio can also assist in electrocardiogram analysis [16]. In the context of fatigue assessment, researchers have predominantly focused on the LFB [14,17] and medium frequency band (MFB) [18]. Fatigue measurements are also being conducted based on calculations of the EMG ratio and power ratio between the HFB (high frequency band) and LFB [6,19].

Esau et al. [20] observed changes in the power spectra of EMG recorded from the skeletal muscles of four healthy adult males following a fatigue activity. Notably, changes in the MPF and HFB were considerably less pronounced compared to those in the H/L (high/low) frequency ratio. Karthick and Ramakrishnan [21] performed EMG assessments through dynamic contractions of the bicep brachii, discovering that the M/LFB was more sensitive to fatigue than the H/LFB. Furthermore, von Tscharner et al. [22] analyzed an EMG of the vastus medialis and vastus lateralis during leg extension movements reporting that the 35–107 Hz band represents the spectrum of most motor units and is particularly sensitive to peripheral fatigue.

Fatigue measurements based on frequency band ratios have predominantly focused on the LFB and HFB; however, recent studies have also explored the use of the MFB in this context [21,23]. Generally, special attention is warranted during fatigue measurements in dynamic situations owing to potential issues resulting from electrode movements and signal instabilities [24,25]. Although MFBbased muscle fatigue measurements have proven effective in dynamic situations [21,23], similar fatigue measurements in static situations or as measures of parameter sensitivity are not recommended. Nevertheless, since parameter performance is less affected by electrode movements and signal stability, it can enable objective evaluations despite the lack of research cases.

Therefore, this study aimed to develop and evaluate new fatigue measurement parameters by creating synthetic parameters, such as H/MFB, (H + M)/LFB, and H/(M + L)FB, which incorporate the MFB known for its high stability in fatigue detection. To compare the fatigue measurement performance of these parameters, EMG data from shoulder muscles were collected, and the performance of the synthetic parameters was evaluated in comparison to existing parameters based on force levels and muscle types. This study seeks to investigate the correlations between the newly introduced parameters, the components of each frequency band, and muscle fatigue, as well as to assess their practical applicability.

## 2. Materials and Methods

### 2.1. Participants

A total of 15 participants (eight males and seven females) with no history of shoulder surgery or pain over the past six months were selected for the EMG measurements. They were all right-handed university students. The average age of the participants was 28.13 (±4.37) years. Furthermore, their average height was 174.47 (±6.56) cm, while their average weight was 70.53 (±8.94) kg. To verify whether the sample size is adequate, a power analysis was conducted using the Power Analysis and Sample Size (PASS) software for simple linear regression analysis at a significance level of 5% [26]. The analysis indicated a power of 0.9240 with a calculated sample size of 7 participants and an R^2^ of 0.6. Considering a dropout rate of 20%, which accounts for 3 participants, the minimum required sample size was determined to be 10. Therefore, a total of 15 participants were recruited, including 5 for a preliminary experiment. This study was approved by the Hanyang University International Review Board (approval No. HYU-2019-04-008) and was conducted in accordance with the Helsinki Declaration as revised in 2008.

### 2.2. Apparatus

**Figure 1:**
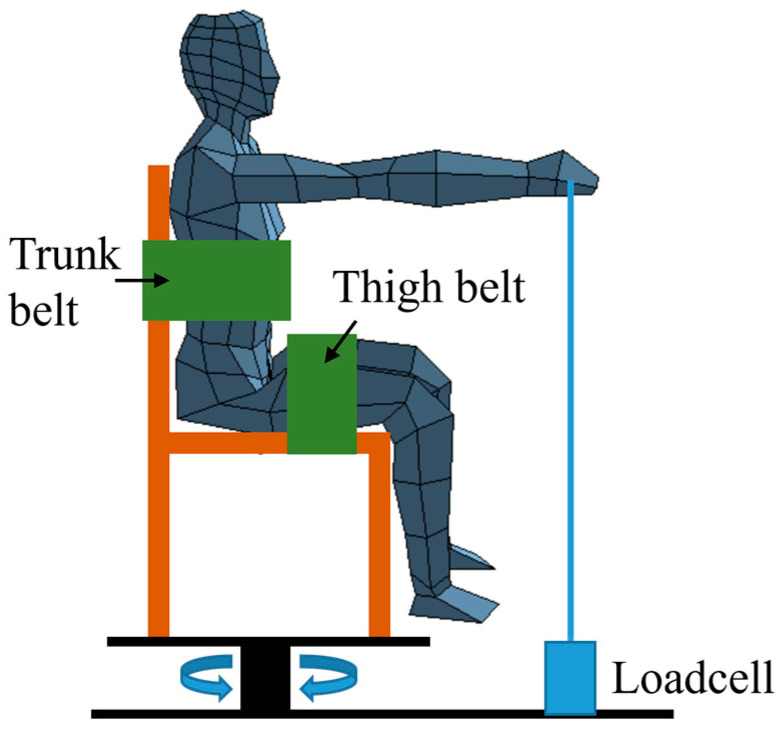
Experimental situation.

A force-measuring sensor (load cell) (Bongsin Co., Seoul, Republic of Korea) capable of measuring forces up to 50 N was employed to measure shoulder muscle strength. The measurement setup included a handle attached to an adjustable-length wire for ease of use by the participants. During posture-based EMG measurements, participants’ bodies and legs were kept stable. Also, the participants were strapped by a cloth belt to the chair, and their feet were kept above the floor, to isolate the upper body muscle. In order to set the shoulder angle in a standardized manner, the acromion and the joint protrusion of the upper arm were used as anatomical reference points and a digital protractor was used to measure the joint angle. Additionally, participants were instructed to pull the force-measuring sensor using only their shoulder muscles and to keep their feet off the ground, ensuring that no additional muscles interfered with the force measurements (Figure 1). Before conducting the experiment, the purpose and precautions of the study were explained to the participants, and they were given approximately half hour to adapt.

An EMG system (Mega Electronics, Kuopio, Finland) was used to record muscle activity using the surface electrode method. The setup included three channels of Ag/AgCl bipolar electrodes with a diameter of 10 mm and an inter-electrode distance of 20 mm and an electrode resistance below 10 Ω. These signals were sampled at a rate of 1000 Hz with a gain of 1000, a common mode rejection ration (CMRR) of 110 dB.

### 2.3. Procudure

During isometric muscle contractions, the force levels and specific muscle types employed by the participants during an arm-lifting activity were considered as independent variables. Furthermore, three predetermined force levels—30%, 40%, and 50% of the maximum voluntary contraction (MVC)—were used. Specifically, 100% MVC tests were conducted three times for each participant, and when the resulting individual measurement values differed by less than ±3 N, the average values were computed, yielding the specific force levels of 30%, 40%, and 50% of the MVC [27]. The shoulder angle and external load were used as independent variables affecting shoulder fatigue. A within-subject design was used experimental conditions consisting of three levels of %MVC including 30%, 40%, and 50% MVC, and shoulder posture including 90 degrees of flexion angle.

**Figure 2:**
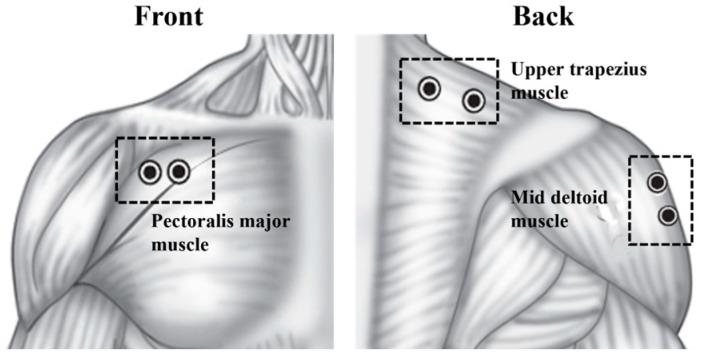
Electrode placements in EMG sensors.

As shown in Figure 2, an experiment was conducted on three muscles to compare the fatigue measurement parameters. These muscles are anatomically and biomechanically associated with the stability of the shoulder complex, shoulder flexion, and arm lifting [28,29,30,31]. Previous studies similar to this study have considered the co-activation between the upper trapezius, mid-deltoid, and pectoralis major muscles [32,33,34,35,36,37]. Following the surface electromyography for non-invasive assessment of muscle (SENIAM) recommendations [38], the skin overlaying the muscle was shaved and cleaned with alcohol gauze to minimized impedance, and electrode locations were marked on the skin with a waterproof pen. Surface electrodes were attached to three major muscles on the right shoulder.

The strength maintenance level and duration for each experimental condition were established based on Rohmert’s curve [39]. For force measurements, MVC testing was performed three times (for 3 s each) at each shoulder posture, and the mean value (34.64 N ± 2.30) was considered the 100% MVC value. Extreme measurement outcomes (±3 N) were treated as outliers. EMG recordings were conducted while participants maintained 30%, 40%, and 50% of their MVCs for 1 min under each posture condition. To maintain each force level, participants monitored and adjusted their force outputs based on the readings displayed on the force transducer. Rest periods of 5 min were allowed between each trial, and the experimental sequence was counterbalanced across the three experiments.

### 2.4. Frequency Band Ratio Parameters

To quantify muscle fatigue under each experimental condition, the initial and final 10 s of the recorded 60-s EMG signal were excluded, yielding a 40-s EMG segment for analysis. Subsequently, the time series values of this segment were converted into frequency values at 1-s intervals using the fast Fourier transform. The frequency bands within the resulting EMG signals were then classified into low (15–45 Hz), medium (46–95 Hz), and high (>95 Hz) bands using MATLAB R2020b (The Mathworks Inc., Natick, MA, USA).

This study developed parameters based on the frequency bands of the electromyographic (EMG) signals. A total of 12 parameters were created by combining high, medium, and low-frequency bands: H/MFB, M/HFB, M/LFB, L/MFB, H/LFB, L/HFB, (H + M)/LFB, L/(H + M)FB, H/(M + L)FB, (M + L)/HFB, (H + L)/MFB, and M/(H + L)FB. The variance of the generated parameters was evaluated using the measured EMG signals. The evaluation results showed that combinations such as M/HFB, L/HFB, L/MFB, L/(H + M)FB, (M + L)/HFB, M/(H + L)FB, and (H + L)/MFB had too large a variance, making them unsuitable for use. In contrast, the combinations H/LFB, M/LFB, H/MFB, (H + M)/LFB, and H/(M + L)FB showed small variances and were stable in evaluating fatigue. The H/LFB and M/LFB parameters have been previously studied by several researchers [18,21]. Therefore, we applied the parameters for fatigue assessment from previous research along with the medium frequency to compare parameters that were not considered in prior studies, such as H/MFB, (H + M)/LFB, and H/(M + L)FB. This was done to identify the characteristics of each parameter. In this study, we aimed to identify parameters that can more effectively evaluate muscle fatigue by applying MFB in various ways with HFB and LFB.

To establish new frequency band parameters, frequency bandwidth ratios were examined. By assessing the combinations of the most commonly employed bands in previous studies using regression analysis—LFB (15–45 Hz), MFB (46–95 Hz), and HFB (>95 Hz)—three promising combinations with large absolute values and low variances were obtained: H/MFB, (H + M)/LFB, and H/(M + L)FB [17,40]. These combinations were defined as synthetic parameters and selected as new parameters for fatigue analysis.

The first parameter was defined as the ratio of HFB to MFB (Equation (1)). It was used as an indicator to evaluate the degree of muscle fatigue.

H/M = HFB/MFB(1)

In previous studies, quantitative muscle fatigue assessments have been conducted based on the premise that reductions in high-frequency ratios lead to increases in low-frequency ratios [6,13,21]. Consequently, HM/L, as defined in Equation (2), was selected as the second parameter to determine whether reductions in the high- and mid-frequency ratios resulting from increases in low-frequency ratios could sensitively indicate fatigue.

HM/L = HFB + MFB/LFB(2)

Similar to the second assumption, the third parameter, H/ML, as defined in Equation (3), was employed to assess whether reductions in the high-frequency ratio resulting from increases in mid- and low-frequency ratios could sensitively reflect fatigue conditions.

H/ML = HFB/MFB + LFB(3)

This study also compared MPF, which generally evaluates muscle fatigue, in addition to suggested parameters [41,42]. Furthermore, the ratios H/LFB, H/MFB, and M/LFB were also selected as comparison parameters [9,13,21]. The fatigue detection performance of the selected fatigue measurement parameters was compared using descriptive statistics and temporal trend analyses. Additionally, we aimed to identify a parameter capable of accurately quantifying fatigue by examining the variations in the selected parameters across different experimental conditions.

### 2.5. Statistics

This study aimed to evaluate muscle fatigue by comparing fatigue measurement parameters based on time-dependent variations through descriptive statistical analysis. The independent variables used for muscle fatigue evaluation included force exertion time (40 s), force levels (30%, 30%, 50% MVCs), and muscle types (upper trapezius, mid-deltoid, and pectoralis major). The dependent variables included MPF, M/LFB, H/MFB, H/LFB, H(M + L)FB, and (H + M)FB. The evaluation of muscle fatigue using the dependent variables was based on the degree of frequency band shift in EMG signals from high-frequency to low-frequency ranges. Previous studies have shown a high correlation between muscle fatigue contractions and force (r = 0.910; *p* < 0.001) [43] and defined muscle fatigue as a decrease in the frequency band by more than 8% toward the low-frequency range [44,45].

To identify significant differences, multivariate analysis of variance (MANOVA) with Tukey’s test for post-hoc comparisons was conducted. Through MANOVA, this study aimed to identify differences in the degree of fatigue among muscles, differences in fatigue levels across force levels, and differences in the sensitivity of each parameter. Each parameter was considered to indicate fatigue when its value decreased by more than 10% of its initial value. Statistically significant parameters were identified through comparisons of fatigue evaluation results, and linear regression analysis was performed to assess the sensitivity of each parameter. The linear regression analysis compared how sensitively the frequency values of each parameter shifted during the force exertion period. All statistical analyses were performed at a significance level of 0.05 using SPSS software v18.0 (Statistical Package for the Social Sciences Inc., Chicago, IL, USA).

## 3. Results

### 3.1. Descriptive Statistics

Table 1 shows the changes in the values of each parameter at 5 s intervals over a 40 s period. The degree of fatigue expression for each parameter was categorized based on the observed reductions in value: 10–19%, 20–29%, and 30% or more. At a 30% MVC force level, the MPF in the mid-deltoid muscle showed a reduction of over 10%. However, no such reduction was observed in the MPF of the pectoralis major at the same force level. These results suggest that the pectoralis major muscle was less active, generating lower forces primarily during adduction movements. A tendency toward fatigue was observed at the 40% and 50% MVC levels. In contrast, no decrease in MPF was noted in the upper trapezius muscle at any force level.

Notably, M/LFB values consistently decreased across all force levels and in all muscles. Furthermore, H/LFB values decreased with increasing force levels in the mid-deltoid, but not in the pectoralis major or upper trapezius muscles. Meanwhile, H/MFB decreased at all force levels in the mid-deltoid and at the 30% MVC level in the upper trapezius; however, no decreasing trend was observed in the pectoralis major, suggesting that this muscle may not effectively reflect fatigue conditions.

Additionally, H/(M + L)FB values decreased at all force levels in both the mid-deltoid and trapezius muscles, while the pectoralis major showed a decreasing trend in these values as force levels increased. At the 30% MVC level, (H + M)/LFB values decreased in the mid-deltoid, whereas in the pectoralis major, these values exhibited a more pronounced decreasing trend with increasing force levels. In contrast, the trapezius muscle showed a decrease in (H + M)/LFB values across all force levels. These findings confirm that each parameter demonstrates a distinct fatigue-indication tendency, depending on the force level and muscle type.

### 3.2. MANOVA

The independent variable used in the MANOVA analysis was the force level, while the dependent variables included fatigue evaluation parameters such as MPF, M/LFB, H/MFB, (H + M)/LFB, and H/(M + L)FB. The MANOVA results showed statistically significant differences (*p* < 0.05) in all parameters, except for (H + M)/LFB, based on %MVC levels. In particular, the H/MFB parameter was found to be significant. Levene’s homogeneity test in SPSS was used to confirm homogeneity at a significance level of α = 0.05. Normality was also tested at a significance level of α = 0.05, followed by MANOVA and post hoc analysis. Additionally, statistically significant differences (*p* < 0.01) were observed across all parameters with respect to force levels (Table 2).

**Figure 3:**
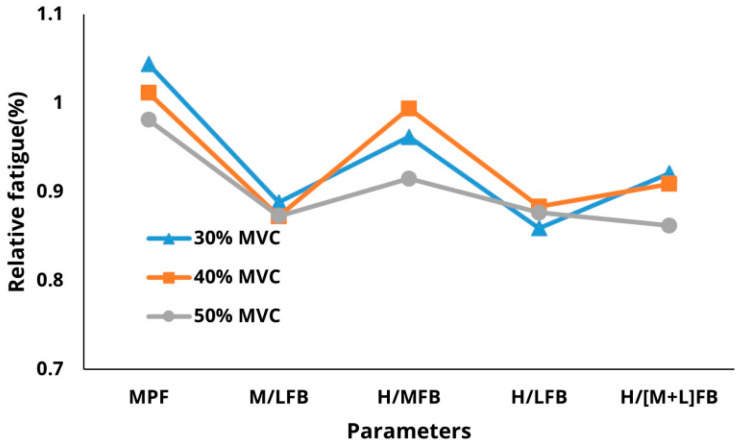
Interactions among fatigue parameters for %MVC levels.

MANOVA was conducted to examine the interaction between parameters and force levels for each treatment. Tukey’s multiple comparison test was conducted as a post-hoc analysis to evaluate the discrimination performance of the fatigue measurement parameters across muscle types and force levels. MPF and H/MFB were classified into 30%, 40%, and 50% MVC fatigue levels, while H/(M + L)FB differentiated between these levels (Figure 3).

**Figure 4:**
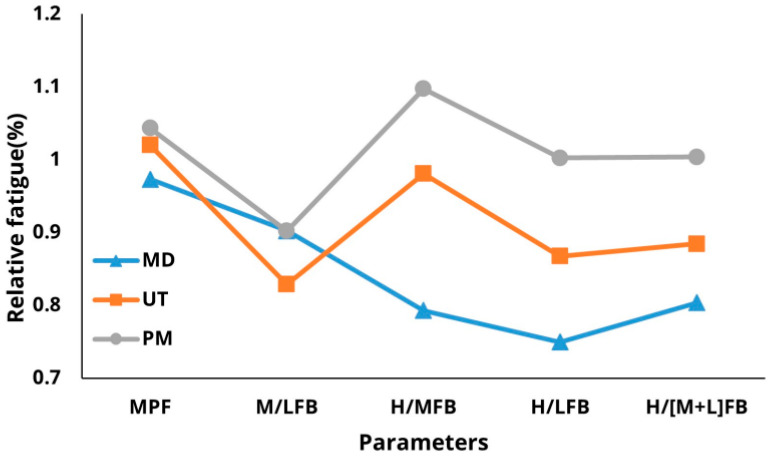
Interactions among fatigue parameters for muscle types.

In terms of muscle position, H/MFB, H/LFB, and H/(M + L)FB effectively classified fatigue across different muscles. The mid-deltoid exhibited the highest relative fatigue, followed by the upper trapezius and pectoralis major, indicating a greater shoulder burden in the mid-deltoid. Additionally, H/MFB and H/(M + L)FB classified fatigue based on both force levels and muscle types, with H/MFB demonstrating superior classification performance compared to H/(M + L)FB. Regarding the interactions among parameters at varying %MVC levels, the interactions between H/LFB and H/(M + L)FB were more pronounced at the 50% MVC level than at the 30% and 40% MVC levels. Moreover, M/LFB, H/LFB, and H/(M + L)FB were better indicators of muscle fatigue compared to other parameters (Figure 4).

### 3.3. Sensitivity of Parameters

To compare the sensitivity of these parameters to fatigue, this study analyzed the standardized coefficients from a linear regression analysis. In the linear regression analysis, elapsed time was used as the independent variable, and the values of each parameter based on muscle type and force level were used as the dependent variables. Residual analysis confirmed that the assumptions of linear regression were satisfied, and the changes in parameters based on muscle type and force level were analyzed using individual regression models. As shown in Table 3, the results of regression analysis revealed generally low standardized regression coefficients for the pectoralis major muscle. This is likely due to the high variability of the parameters and the limited involvement of the pectoralis major in arm adduction and abduction movements, leading to reduced muscle use and, consequently, lower fatigue levels. Additionally, as the %MVC level increased, the slope of H/(M + L)FB also increased.

The mid-deltoid muscle exhibited extremely high sensitivity, with standardized regression coefficients of −0.915 for H/MFB, −0.931 for H/LFB, and −0.943 for H/(M + L)FB. In contrast, the pectoralis major muscle showed positive standardized regression coefficients for all parameters, indicating no decrease in relative fatigue. The upper trapezius also demonstrated high sensitivity, with standardized regression coefficients of −0.7 or higher for all parameters except H/MFB (−0.586), and the highest coefficient of −0.874 for H/LFB. Consequently, fatigue was confirmed to occur only in the mid-deltoid and upper trapezius muscles at 30% MVC.

At the 40% MVC level, the mid-deltoid muscle exhibited high sensitivity, with a standardized regression coefficient of −0.924 for H/MFB and −0.848 for H/LFB. The pectoralis major muscle also showed high sensitivity, with a coefficient of −0.820 for H/(M + L)FB. Additionally, the upper trapezius muscle demonstrated particularly high sensitivity, with standardized regression coefficients of −0.902 for M/LFB and −0.960 for H/(M + L)FB.

At the 50% MVC level, the mid-deltoid muscle demonstrated high sensitivity, with standardized regression coefficients of −0.887 for H/MFB, −0.717 for H/LFB, and −0.802 for H/(M + L)FB. The pectoralis major muscle also showed high sensitivity, with coefficients of −0.802 for M/LFB and −0.797 for H/(M + L)FB. Additionally, the upper trapezius muscle exhibited particularly high sensitivity, with standardized regression coefficients of −0.929 for M/LFB and −0.924 for H/(M + L)FB. These results indicate that M/LFB and H/(M + L)FB are relatively more sensitive to temporal changes in fatigue in both the mid-deltoid and upper trapezius muscles.

## 4. Discussion

Fatigue parameters based on frequency band ratios have been a focal point of research in EMG based fatigue assessments, with extensive analysis of their strengths and limitations. Allison and Fujiwara [6] reported that the H/LFB ratio is particularly valued as a quantitative and practical measure of muscle fatigue, as it reflects increases in low-frequency ratios accompanied by reductions in high-frequency component ratios. Frequency band ratio measurements have proven effective in assessing muscle fatigue under specific experimental conditions. Karthick and Ramakrishnan [21] categorized the frequency band of surface EMG signals into three groups: LFB (15–45 Hz), MFB (55–95 Hz), and HFB (>95 Hz).

In this study, low, medium, and high frequencies were analyzed, and the results revealed that H/(M + L)FB was more sensitive to fatigue than (H + M)/LFB. This finding suggests that the MFB exhibits a behavior similar to that of the LFB parameter. In the mid-deltoid and upper trapezius muscles, the LFB and MFB values tended to gradually increase over time. We found that the frequency ratio of LFB and MFB increased under fatigue conditions. Thus, comparing the behaviors of LFB and MFB with that of HFB provides a more sensitive evaluation of fatigue.

Table 4 summarizes the fatigue patterns of six parameters. Although the MPF parameter tends to underestimate fatigue conditions compared to other parameters, it still effectively distinguishes fatigue based on force levels. These findings could be attributed to the high correlation between spectral change methods and the force levels of surface EMG signals [1]. While M/LFB is closely related to MPF, it may exhibit reduced sensitivity under conditions of significant muscle fatigue and decreased muscle strength [6]. Additionally, Karthick and Ramakrishnan [21] reported that M/LFB is more sensitive to fatigue than H/LFB.

H/LFB effectively reflected initial fatigue conditions and distinguished between fatigue conditions based on muscle types. The (H + M)/LFB index effectively represented the fatigue of the pectoralis major and upper trapezius muscles but failed to accurately reflect the fatigue of the mid-deltoid muscle. This phenomenon is interpreted as a result that, similar to MPF, is not sensitive to severe muscle fatigue and strength reduction. Additionally, the H/(M + L)FB parameter tended to underestimate fatigue but provided a more stable evaluation compared to other parameters.

Comparisons among the sensitivity levels of the six parameters showed that H/LFB, M/LFB, and H/(M + L)FB were sensitive to fatigue conditions. The effectiveness of these parameters in detecting fatigue varied based on experimental conditions. H/MFB was found to effectively distinguish fatigue levels based on force. However, it showed sensitivity to fatigue only in the mid-deltoid and did not seem to adequately represent fatigue levels in the upper trapezius and pectoralis major muscles. MPF, H/LFB, and H/(M + L)FB were more sensitive to the degree of fatigue, while M/LFB was less effective in indicating fatigue levels. Notably, the MPF demonstrated the poorest fatigue detection performance among all parameters across all experimental conditions. These findings suggest that MPF and M/LFB ratios are less accurate in reflecting fatigue conditions under varying force levels.

Comparisons between parameters according to the muscle type revealed that the MPF, M/LFB, H/MFB, H/(M + L)FB, and (H + M)/LFB differentiated between fatigue conditions. Most parameters detected fatigue in the mid-deltoid and upper trapezius muscles, confirming the result indicating greater fatigue levels in the mid-deltoid and trapezius muscles in postures where the arm is raised above the shoulder [46,47]. However, the parameters were not as effective in detecting fatigue in the pectoralis major muscle, likely owing to the predominant involvement of this muscle in adduction or internal rotation of the humerus, which results in reduced muscle usage [48].

Differences among parameters based on force levels indicated that MPF could not effectively distinguish fatigue levels between 30% and 40% MVC, but it clearly distinguished the differences in fatigue levels at 50% MVC. These results suggest that the sensitivity of MPF is not high at low force levels when assessing fatigue through repeated EMG measurements [6]. On the other hand, the H/MFB, H/LFB, and H/(M + L)FB parameters effectively distinguished fatigue levels based on %MVC, whereas M/LFB did not. Cardozo et al. [49] reported that frequency band ratios are more sensitive to fatigue conditions compared to MPF and can, therefore, serve as valuable indicators for fatigue assessment. In this context, methods based on frequency spectrum distributions, such as the H/(M + L)FB used in this study, consistently demonstrate better performance in evaluating the relative changes in muscle fiber conduction velocity compared to MPF [23].

Compared to other parameters, the H/LFB values showed significant differences depending on experimental conditions, with a notable decreasing trend at higher force levels. This pattern is consistent with the findings of Gross et al. [11], who demonstrated that the variation rate and magnitude of the H/LFB parameter are influenced by the initial force value. The results of this study confirmed that the sensitivities of the M/LFB and H/(M + L)FB parameters increased with greater muscle exertion, suggesting that these ratios can provide insights into force-related metrics, such as the RMS value. However, the M/LFB parameter was less responsive to initial fatigue, as indicated by its minimal response at the 50% MVC level and its very small standardized regression coefficient. The H/(M + L)FB index showed limited sensitivity to early fatigue but improved after 20 s. These results suggest that both indices fail to reflect muscle fatigue during initial muscle use but begin to capture the effects of strength reduction caused by sustained muscle activity. Mesin et al. [1] stated that the spectral index, median frequency (MF), is influenced by both conduction velocity (CV) and motor unit (MU) synchronization, limiting its ability to distinguish peripheral fatigue. Since H/(M + L)FB is also a spectral index, it is expected to share similar limitations in identifying peripheral fatigue. To address these limitations, future studies should compare it with CV to assess the sensitivity of spectral indices. Furthermore, as %MVC levels increased, the fatigue sensitivities of both parameters improved. This aligns with Karthick and Ramakrishnan [21], who found reductions in the proportion of medium frequencies with increasing muscle forces.

A review of the performance outcomes for all parameters confirmed that the fatigue sensitivities of H/(M + L)FB and M/LFB, which incorporate MFB information, are superior to those of other parameters. Based on these observations, we recommend using parameters that account for all EMG band ratios for accurate muscle fatigue assessments. However, since the sensitivity of parameters like MFB tends to slightly deteriorate at higher force levels, these parameters should be used with caution and subjected to thorough examination of MFB dynamics.

Based on the fatigue measurement results, the response sensitivities of the H/LFB, M/LFB, and H/(M + L)FB parameters were evaluated. The results revealed that the H/LFB parameter was sensitive to temporal changes under all test conditions, with a high relative fatigue rate of 107–175% compared to the MPF, indicating its potential as a good indicator of fatigue. However, as reported by Moxham et al. [24], there was significant variability in the H/LFB parameter below the 30% MVC level. Thus, despite its notable fatigue sensitivity, the effectiveness and accuracy of H/LFB appear to be limited. The H/(M + L)FB parameter also demonstrated sensitivity to temporal changes across all conditions. These results suggest that, at low force levels, the H/(M + L)FB parameter better represents fatigue progression over time compared to other parameters.

Based on results of this study, we analyzed the characteristics of the fatigue measurement parameters and identified optimal measurement conditions. Among the introduced parameters, H/LFB effectively classified fatigue according to the muscle type and was found to be particularly advantageous for quantifying fatigue resulting from activities with short cycle times and isotonic muscle contractions [6]. However, the considerable variability of this parameter at low force levels must be considered during fatigue measurements.

The H/(M + L)FB parameter distinguished fatigue conditions based on force levels and muscle types, responding well to temporal changes under all conditions. Similar to M/LFB, the H/(M + L)FB parameter was less affected by the reductions in muscle strength observed during repeated experiments, demonstrating a relative fatigue rate of 107–150% compared to the MPF. While this parameter proved effective in fatigue detection, its fatigue detection accuracy deteriorated with increasing force levels. Hence, fatigue assessments using this parameter must involve thorough examinations of MFB dynamics.

The H/(M + L)FB parameter proposed in this study is particularly suitable for fatigue measurements among industry workers who are often engaged in work requiring low force levels but long working hours. This parameter can be used for fatigue evaluations among workers engaged in electronics and automobile manufacturing, food and lodging, and consumer product repair industries, where characteristic tasks do not typically involve heavy lifting but require repetitive hand usage. Conversely, the H/LFB parameter is more suitable for fatigue assessments among industry workers who frequently handle heavy goods and work in short durations. Thus, the H/LFB parameter is better suited for fatigue assessments in the ship manufacturing, construction, building management, health or social welfare, and medical support industries.

## 5. Conclusions

This study developed fatigue measurement parameters based on frequency band ratios. Comprehensive evaluations revealed that the H/LFB parameter, introduced in previous research, and the H/(M + L)FB parameter, introduced in this study, exhibited high sensitivity to fatigue across various conditions. Notably, while the H/LFB parameter showed significant variability at low force levels, it effectively detected fatigue across different muscle types at high force levels. In contrast, the H/(M + L)FB parameter reliably detected fatigue in various muscles even at low force levels.

Therefore, we recommend using the H/(M + L)FB parameter for fatigue assessments in activities involving lower force levels, and the H/LFB parameter for environments requiring higher force levels. To further enhance the reliability of these parameters, future research could expand to include additional muscle types and conditions. The parameters introduced in this study are anticipated to be valuable indices for accurate shoulder fatigue assessments in static states across different working environments. Moreover, the accuracy and practical applicability of these parameters could be improved by incorporating isotonic or dynamic contraction tasks and evaluating additional muscle groups.

## Figures and Tables

**Table 1:** Regression results of reductions in fatigue ratios using parameters (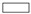 indicates conditions with a <10% decrease in each parameter value; 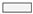 indicates conditions with a 10–19% reduction in each parameter value; 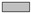 indicates conditions with a 20–29% decrease in each parameter value; and 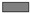 indicates conditions with a ≥30% decrease in each parameter value).

Muscle	%MVC	Parameter	Time (s)								Mean (SD)
			5	10	15	20	25	30	35	40	
Mid-deltoid	30%	MPF	0.93	0.97	1.01	0.92	0.99	0.99	0.91	0.86	0.97 (0.04)
		M/LFB	0.87	0.85	0.88	0.82	0.73	0.77	0.77	0.61	0.80 (0.08)
		H/MFB	1.08	0.93	0.88	0.90	0.87	0.80	0.75	0.79	0.89 (0.12)
		H/LFB	0.94	0.80	0.78	0.74	0.64	0.62	0.57	0.48	0.72 (0.14)
		H/(M + L)/FB	1.03	0.88	0.84	0.84	0.78	0.73	0.68	0.65	0.82 (0.13)
		(H + M)/LFB	0.89	0.84	0.86	0.80	0.71	0.74	0.72	0.58	0.78 (0.09)
	40%	MPF	1.02	1.11	1.06	1.21	1.24	1.26	1.31	1.31	0.96 (0.08)
		M/LFB	1.01	0.92	0.94	0.89	0.96	0.90	0.90	0.89	0.81 (0.07)
		H/MFB	0.87	0.70	0.66	0.62	0.58	0.53	0.51	0.50	0.83 (0.13)
		H/LFB	1.00	0.84	0.78	0.82	0.74	0.73	0.71	0.71	0.67 (0.14)
		H/(M + L)/FB	0.92	1.11	1.06	0.97	0.93	1.06	0.98	0.85	0.76 (0.13)
		(H + M)/LFB	1.07	1.17	1.12	1.11	1.16	1.04	1.23	1.49	0.78 (0.08)
	50%	MPF	0.96	1.05	1.08	1.14	1.14	1.12	1.10	1.19	0.97 (0.08)
		M/LFB	0.99	0.92	0.95	0.88	0.96	0.88	0.90	1.16	0.96 (0.09)
		H/MFB	0.91	0.82	0.73	0.74	0.54	0.69	0.59	0.52	0.72 (0.11)
		H/LFB	1.01	0.89	0.76	0.76	0.72	0.87	0.71	0.77	0.69 (0.14)
		H/(M + L)/FB	0.75	0.90	0.77	0.74	0.69	0.60	0.60	0.65	0.71 (0.12)
		(H + M)/LFB	0.91	0.84	0.89	0.92	0.76	0.85	0.76	1.09	0.90 (0.10)
Pectoralis major	30%	MPF	0.89	0.98	1.10	1.16	1.18	1.21	1.36	1.38	1.13 (0.14)
		M/LFB	0.99	0.95	0.99	1.04	1.12	0.98	1.06	1.07	1.02 (0.06)
		H/MFB	0.94	1.11	0.98	0.89	1.04	1.19	1.08	1.098	1.04 (0.11)
		H/LFB	0.93	1.06	0.96	0.93	1.17	1.16	1.14	1.17	1.06 (0.12)
		H/(M + L)/FB	0.93	1.08	0.97	0.91	1.11	1.17	1.12	1.13	1.05 (0.11)
		(H + M)/LFB	0.98	0.97	0.98	1.02	1.13	1.00	1.07	1.08	1.02 (0.06)
	40%	MPF	1.01	0.92	0.94	0.89	0.96	0.90	0.90	0.89	1.09 (0.10)
		M/LFB	0.92	1.11	1.06	0.97	0.93	1.06	0.98	0.85	1.04 (0.07)
		H/MFB	1.07	1.17	1.12	1.11	1.16	1.04	1.23	1.49	1.15 (0.12)
		H/LFB	0.93	0.92	0.95	1.03	0.98	0.93	1.07	0.94	1.19 (0.13)
		H/(M + L)/FB	0.83	0.74	0.72	0.74	0.73	0.81	0.68	0.71	1.18 (0.12)
		(H + M)/LFB	1.04	0.83	0.82	0.80	0.83	0.81	0.81	0.81	1.06 (0.07)
	50%	MPF	0.99	0.92	0.95	0.88	0.96	0.88	0.90	1.16	0.99 (0.10)
		M/LFB	0.75	0.90	0.77	0.74	0.69	0.60	0.60	0.65	0.91 (0.07)
		H/MFB	0.91	0.84	0.89	0.92	0.76	0.85	0.76	1.09	0.90 (0.08)
		H/LFB	0.85	0.90	0.85	0.87	1.06	0.97	0.96	1.27	0.82 (0.08)
		H/(M + L)/FB	1.04	0.96	0.94	0.92	0.78	0.91	0.84	0.79	0.93 (0.07)
		(H + M)/LFB	1.02	0.93	0.87	0.87	0.83	0.82	0.73	0.83	0.90 (0.07)
Upper trapezius	30%	MPF	0.92	1.00	1.06	0.93	0.97	1.01	1.04	1.10	1.01 (0.05)
		M/LFB	1.05	0.89	0.96	0.88	0.87	0.85	0.84	0.80	0.93 (0.10)
		H/MFB	1.01	1.07	0.99	0.90	0.91	0.85	0.89	0.85	0.93 (0.06)
		H/LFB	1.06	0.95	0.95	0.79	0.79	0.73	0.75	0.68	0.87 (0.12)
		H/(M + L)/FB	1.03	1.03	0.97	0.86	0.86	0.81	0.84	0.79	0.91 (0.08)
		(H + M)/LFB	1.05	0.91	0.96	0.86	0.85	0.82	0.82	0.77	0.91 (0.10)
	40%	MPF	0.93	0.92	0.95	1.03	0.98	0.93	1.07	0.94	1.17 (0.11)
		M/LFB	0.96	0.82	0.73	0.70	0.70	0.71	0.69	0.65	0.87 (0.08)
		H/MFB	0.97	1.00	1.02	1.07	1.04	1.03	0.91	1.06	0.94 (0.05)
		H/LFB	1.09	1.00	0.98	0.86	0.99	0.97	0.84	0.94	0.81 (0.09)
		H/(M + L)/FB	0.98	0.83	0.80	0.72	0.68	0.56	0.61	0.58	0.89 (0.06)
		(H + M)/LFB	0.96	0.96	0.88	0.95	0.82	0.82	0.80	0.79	0.85 (0.08)
	50%	MPF	0.85	0.90	0.85	0.87	1.06	0.97	0.96	1.27	1.09 (0.06)
		M/LFB	0.95	0.82	0.84	0.84	0.74	0.67	0.67	0.62	0.88 (0.11)
		H/MFB	0.89	0.93	0.90	1.01	0.98	0.95	1.07	0.80	0.92 (0.07)
		H/LFB	1.17	1.03	1.12	1.01	0.91	0.91	0.94	0.91	0.80 (0.10)
		H/(M + L)/FB	0.87	0.82	0.73	0.75	0.59	0.70	0.62	0.57	0.88 (0.07)
		(H + M)/LFB	1.00	0.93	0.81	0.81	0.80	0.99	0.87	0.85	0.86 (0.10)

**Table 2:** The MANOVA results for the fatigue detection parameters between the force levels and muscle types.

Source	Variables	SS	*df*	MS	*F*	*p*
%MVC	MPF	0.709	2	0.354	63.206	0.001
	M/LFB	0.057	2	0.028	3.239	0.040
	H/MFB	1.141	2	0.570	45.684	0.001
	H/LFB	0.111	2	0.056	3.328	0.036
	H/(M + L)FB	0.698	2	0.349	27.610	0.001
	(H + M)/LFB	0.020	2	0.010	1.124	0.326
Muscle	MPF	0.920	2	0.460	82.006	0.001
	M/LFB	1.300	2	0.650	74.062	0.001
	H/MFB	16.966	2	8.483	679.477	0.001
	H/LFB	11.543	2	5.772	345.082	0.001
	H/(M + L)FB	7.282	2	3.641	287.965	0.001
	(H + M)/LFB	0.712	2	0.356	39.308	0.001
%MVC × Muscle	MPF	0.728	4	0.182	32.447	0.001
	M/LFB	2.395	4	0.599	68.198	0.001
	H/MFB	2.222	4	0.556	44.498	0.001
	H/LFB	0.377	4	0.094	5.639	0.001
	H/(M + L)FB	0.536	4	0.134	10.589	0.001
	(H + M)/LFB	2.302	4	0.576	63.531	0.001

**Table 3:** The regression results for the %MVC levels.

%MVC	Muscle	Parameters	Slope	Intercept	Standardized Coefficients	*R* ^2^	95% CI	
							Low	High
30% MVC	Mid- deltoid	M/LFB	−0.004	0.895	−0.694	0.481	0.895	0.932
		H/MFB	−0.009	1.079	−0.915	0.837	1.047	1.111
		H/LFB	−0.011	0.953	−0.931	0.866	0.918	0.988
		H/(M + L)FB	−0.010	1.034	−0.943	0.890	1.006	0.062
	Pectoralis major	M/LFB	0.002	0.985	0.302	0.091	0.948	1.022
		H/MFB	0.006	0.927	0.626	0.392	0.872	0.982
		H/LFB	0.007	0.910	0.722	0.521	0.895	0.965
		H/(M + L)FB	0.007	0.916	0.710	0.504	0.865	0.968
	Upper trapezius	M/LFB	−0.006	1.059	−0.775	0.601	1.019	1.099
		H/MFB	−0.003	0.999	−0.586	0.343	0.965	1.033
		H/LFB	−0.009	1.051	−0.874	0.765	1.013	1.089
		H/(M + L)FB	−0.005	1.015	−0.797	0.635	0.985	1.045
40% MVC	Mid- deltoid	M/LFB	−0.004	1.049	−0.531	0.282	1.003	1.095
		H/MFB	−0.010	1.035	−0.924	0.854	1.003	1.067
		H/LFB	−0.006	0.945	−0.848	0.718	0.914	0.977
		H/(M + L)FB	−0.005	1.071	−0.528	0.279	1.015	1.127
	Pectoralis major	M/LFB	−0.005	1.071	−0.528	0.279	1.015	1.127
		H/MFB	0.007	1.015	0.627	0.393	0.952	1.079
		H/LFB	0.001	0.947	0.302	0.091	0.912	0.982
		H/(M + L)FB	−0.005	0.889	−0.820	0.672	0.859	0.918
	Upper trapezius	M/LFB	−0.009	0.951	−0.902	0.814	0.919	0.983
		H/MFB	−0.003	0.999	−0.663	0.440	0.973	1.026
		H/LFB	−0.005	1.104	−0.690	0.477	1.062	1.145
		H/(M + L)FB	−0.011	0.983	−0.960	0.921	0.958	1.007
50% MVC	Mid- deltoid	M/LFB	−0.002	0.994	−0.217	0.047	0.941	1.047
		H/MFB	−0.008	0.886	−0.887	0.787	0.853	0.919
		H/LFB	−0.006	0.936	−0.717	0.514	0.888	0.985
		H/(M + L)FB	−0.007	0.889	−0.802	0.643	0.848	0.930
	Pectoralis major	M/LFB	−0.007	0.889	−0.802	0.643	0.848	0.93
		H/MFB	0.001	0.891	0.050	0.003	0.836	0.946
		H/LFB	0.004	0.863	0.552	0.305	0.814	0.913
		H/(M + L)FB	−0.007	1.033	−0.797	0.636	0.994	1.071
	Upper trapezius	M/LFB	−0.010	0.976	−0.929	0.863	0.946	1.005
		H/MFB	−0.001	0.935	−0.144	0.021	0.892	0.979
		H/LFB	−0.006	1.142	−0.727	0.528	1.098	1.186
		H/(M + L)FB	−0.010	0.903	−0.924	0.853	0.872	0.934

**Table 4:** Summary of fatigue patterns for each parameter; N/A: parameters decreased by less than 10%. Slightly decreased: parameters decreased by 10% to less than 20%. Decreased: parameters decreased by more than 20%.

Muscle	%MVC	MPF	M/LFB	H/LFB	H/MFB	H/(M + L)FB	(H + M)/LFB
Mid- deltoid	30%	Decreased	Decreased	Decreased	Decreased	Decreased	Decreased
	40%	N/A	Decreased	Decreased	Decreased	Slightly decreased	N/A
	50%	N/A	Decreased	Decreased	Decreased	Decreased	N/A
Pectoralis major	30%	N/A	N/A	N/A	N/A	N/A	N/A
	40%	Decreased	Decreased	N/A	N/A	Decreased	Decreased
	50%	N/A	Decreased	N/A	N/A	Decreased	Decreased
Upper trapezius	30%	N/A	Decreased	Decreased	Decreased	Decreased	Decreased
	40%	Slightly decreased	Decreased	N/A	N/A	Decreased	Decreased
	50%	Slightly decreased	Decreased	N/A	N/A	Decreased	Decreased

## Data Availability

The original contributions presented in this study are included in the article. Further inquiries can be directed to the corresponding author.
